# Study on salient object segmentation based on depth information guidance and SAM low-rank adaptation fine-tuning

**DOI:** 10.1371/journal.pone.0340765

**Published:** 2026-01-23

**Authors:** Weiping M.A.

**Affiliations:** 1 College of Electronics and Communication Engineering, Lanzhou university of arts and science, Lanzhou, Gansu, China; Chongqing Normal University, CHINA

## Abstract

Accurate segmentation of salient objects is crucial for various computer vision applications including image editing, autonomous driving, and object detection. While research on using depth information (RGB-D) in saliency detection is gaining significant attention, its broad application is limited by dependencies on depth sensors and the challenge of effectively integrating RGB and depth information. To address these issues, we propose an innovative method for salient object segmentation that integrates the Segment Anything Model (SAM), depth information, and cross-modal attention mechanisms. Our approach leverages SAM for robust feature extraction and combines it with a pre-trained depth estimation network to capture geometric information. By dynamically fusing features from RGB and depth modalities through a cross-modal attention mechanism, our method enhances the ability to handle diverse scenes. Additionally, we achieve computational efficiency without compromising precision by employing lightweight LoRA fine-tuning and freezing pre-trained weights. The use of a UNet decoder refines the segmentation output, ensuring the preservation of target boundary details in high-resolution outputs. Experiments conducted on five challenging benchmark datasets validate the effectiveness of our proposed method. Results show significant improvements over existing methods across key evaluation metrics, including MaxF, MAE, and S-measure. Particularly in tasks involving complex backgrounds, small targets, and multiple salient object segmentation, our method demonstrates superior performance and robustness. The significance of this work lies in advancing the application of depth-guided RGB in salient object segmentation while offering new insights into overcoming depth sensor dependency. Furthermore, it opens up novel pathways for the effective fusion of cross-modal information, thereby contributing to the broader development and diversification of related technologies and their applications.

## 1. Introduction

Salient object segmentation is a key task in the field of computer vision, aiming to accurately identify the most attention-grabbing regions or objects in images or videos [1]. In recent years, with the rapid development of deep learning technologies, especially the introduction of convolutional neural networks (CNNs) and self-attention mechanisms, significant progress has been made in salient object segmentation research [2–4]. However, challenges such as complex scenes, multi-modal fusion, multiple objects, and small targets still require further solutions.

Before the rise of deep learning technologies, salient object segmentation primarily relied on hand-crafted feature extraction algorithms. These traditional methods, such as saliency detection algorithms based on color, brightness, texture, and contrast, could achieve certain results under specific conditions but generally had limited capability in handling complex compositions, dynamic scenes, and occluded objects [5–8]. Due to their inability to capture deep semantic information from images, these methods performed less effectively in complex environments. For instance, distinguishing and identifying salient regions in conditions like strong lighting, shadows, or richly colored backgrounds often resulted in misjudgments.

With the development of deep learning, CNN-based models have begun to dominate the task of salient object segmentation [3]. For example, representative models like CPD [9] and MINet [10] introduced multi-scale feature fusion and context information through end-to-end training, improving segmentation accuracy. However, these models typically focus on local features and their reliance on annotated data limits their performance in data-scarce environments [11–13]. They also lack effective adaptability in complex environments and for small targets.

In recent years, the application of Transformers and their variants in image processing has gradually increased. Vision Transformers (ViT) [14] capture global dependencies in images effectively through self-attention mechanisms, demonstrating better performance across various visual tasks compared to CNN models. These Transformer models, such as DINOv2 [15], exhibit good generalization capabilities after large-scale pre-training [16–18]. To enhance computational efficiency and feature extraction in Transformer models, many new studies have introduced windowed self-attention mechanisms and domain-specific optimizations, like Swin Transformer [19] and MViT [20]. These models dynamically adjust spatial resolution at different layers, reducing computational costs while enhancing the fineness of feature extraction.

Depth information, as an important auxiliary modality, provides geometric structure information of three-dimensional space, helping models better distinguish salient objects from the background. Research shows that traditional RGB-D salient object segmentation methods, which integrate RGB and sensor-acquired depth information, significantly improve model robustness when handling complex scenes, occlusions, and dynamic lighting changes [21–24]. For instance, methods like Depth-aware Saliency Detection enhance spatial understanding of objects through depth information, markedly improving segmentation performance. However, these methods typically depend on depth sensors, limiting their applicability in single-modal RGB image scenarios. Additionally, how to efficiently fuse RGB images and depth maps, dynamically balancing the feature weights of both modalities, remains a significant challenge in current research.

Despite the progress made by existing salient object segmentation methods in static images and simple scenes, most are limited to processing single-modal information and fail to fully integrate RGB images with depth information. Consequently, they perform poorly in the presence of complex backgrounds, occlusions, lighting changes, multiple objects, and small target detection, struggling to simultaneously capture the spatial geometric structure and semantic features of objects. To address these issues, this study proposes a method that combines the Segment Anything Model (SAM) backbone network, depth information extraction, and a cross-modal cross-attention mechanism. This method enhances feature representation by introducing depth information to supplement regions that are difficult to accurately distinguish based solely on RGB features. It adopts a low-rank adaptation (LoRA) strategy to fine-tune partial parameters of the SAM2 backbone network, retaining the advantages of pre-trained models while avoiding overfitting risks. The cross-modal cross-attention mechanism dynamically adjusts the weights of information from different modalities, balancing the spatial geometric structure and saliency semantic information of objects. Based on the UNet architecture, it integrates high-resolution low-level features to ensure that detail information is not lost during upsampling, achieving high-precision segmentation. This approach not only improves the accuracy of salient object segmentation but also enhances the flexibility and robustness of the model, making it suitable for various complex scenarios. Superior performance has been demonstrated on multiple benchmark datasets, providing more reliable segmentation results. Our primary contribution lies in proposing an effective and efficient framework for adapting large-scale models (SAM) to the task of salient object segmentation, demonstrating how internally estimated depth, guided by a cross-modal attention mechanism and optimized with LoRA, can significantly enhance performance without requiring external depth sensors.

## 2. Methodology

### 2.1. Overall model architecture

**Fig 1:**
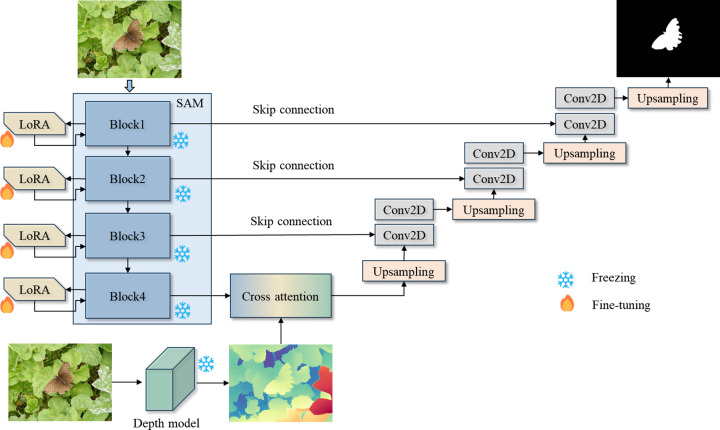
Overall framework of the method.

This study introduces an innovative salient object segmentation method that integrates the Segment Anything Model 2 (SAM2) backbone, depth information, and a cross-modal cross-attention mechanism to achieve efficient and high-precision segmentation. The overall architecture of the model includes five key components: the SAM2 backbone network, low-rank adaptation fine-tuning (LoRA), depth information extraction, cross-modal cross-attention fusion, and a UNet decoding structure with progressive upsampling, as shown in Fig 1.

In this framework, the input image is first processed through the SAM2 backbone for robust feature extraction, generating high-dimensional feature representations. Concurrently, the same image is fed into a depth information extraction module, which leverages a pre-trained depth estimation network to supplement spatial information and object details. Both the SAM2 backbone and the depth extraction network utilize frozen pre-trained weights from large-scale datasets, ensuring stability and generalizability. To adapt the SAM2 backbone to salient object datasets, lightweight LoRA fine-tuning is applied, enabling efficient transfer learning. The feature fusion module employs a cross-modal cross-attention mechanism to seamlessly integrate RGB and depth features, enhancing the model’s ability to handle diverse scenes.

During the decoding phase, a UNet-based structure progressively restores spatial resolution through upsampling operations, ultimately producing high-quality saliency maps. This approach ensures that target boundary details are preserved in high-resolution outputs, contributing to more accurate and reliable segmentation results.

### 2.2. SAM and LoRA

SAM excels in the field of image segmentation and has become a powerful starting point for various image segmentation tasks [25–27]. SAM 2 is an optimized version of SAM, trained on a broader dataset and supporting video feature extraction. Trained on 11 million images and 11 billion masks, SAM 2 exhibits excellent zero-shot transfer capabilities, enabling it to work effectively on unseen data. This makes it a robust feature extractor for salient object segmentation.

**Fig 2:**
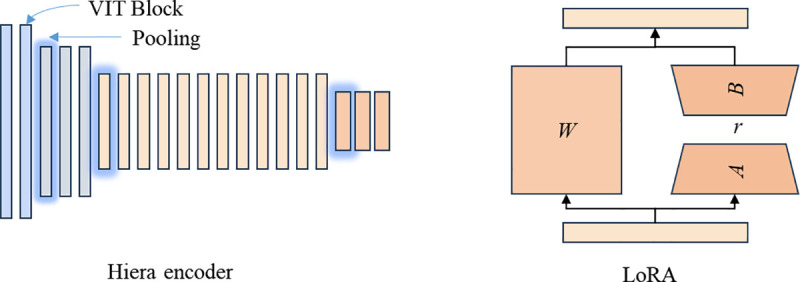
Backbone network and LoRA fine-tuning method applied in SAM2.

As shown in Fig 2, SAM 2 employs a Hiera encoder with multi-scale feature extraction capabilities. Unlike traditional vision transformers (such as ViT) that maintain consistent spatial resolution and feature quantity throughout the network, Hiera optimizes efficiency by using fewer features in early layers and reducing spatial resolution in later layers. Unlike Swin or MViT, which enhance model performance through the introduction of complex modules, Hiera achieves higher efficiency and better performance in multiple image and video recognition tasks by pre-training with a masked autoencoder and removing unnecessary modules. More importantly, the Hiera encoder can generate features at different scales, which is particularly critical for saliency segmentation tasks that require both detail retention and semantic understanding.

SAM2 achieves strong representation capabilities through large-scale pre-training. However, directly fine-tuning all parameters of SAM2 might lead to overfitting or catastrophic forgetting of the general representations learned from large datasets. Therefore, we adopt a method that combines freezing backbone weights with low-rank adaptation (LoRA) for lightweight fine-tuning [28]. By keeping the weights of the SAM2 backbone network unchanged, we ensure that the robust features learned during large-scale pre-training are retained. We introduce low-rank matrices as trainable parameters in the attention layers of SAM2. This approach significantly reduces the computational and memory costs of fine-tuning while effectively capturing task-specific features. The LoRA update formula is as follows:


$${𝐖}_{LoRA}=𝐀{𝐁}^{T}$$ (1)

Where, $𝐀\in R^{r\times d}$ and $𝐁\in R^{d\times r}$are low−rank matrices, and r<<d, reducing the number of parameters during the fine-tuning process.

By fine-tuning with only a few parameters, we can effectively capture task-specific features during training while maintaining lower resource consumption. This strategy not only fully leverages the powerful characteristics of SAM2 but also preserves high generalization ability. This approach, by drastically reducing the number of trainable parameters compared to full fine-tuning, inherently acts as a strong regularizer. It mitigates the risk of overfitting on the smaller downstream datasets while efficiently adapting the model to the specific task, all while retaining the powerful, general-purpose features learned by the frozen backbone.

### 2.3. Dual-path cross-attention network for depth information extraction

Depth information is crucial for understanding the geometric structure and spatial relationships of objects, especially in complex scenes. In this study, we introduce depth estimation information to enhance the model’s expressiveness. Depth estimation employs the Depth Anything V2 network, a robust depth estimation network that excels in extracting depth information. This strategy allows our model to leverage geometric cues without requiring specialized RGB-D hardware, thereby addressing a key limitation of traditional RGB-D models and broadening the applicability of depth-guided segmentation. Subsequently, the extracted depth features are processed using convolutional encoding and activation function modules to generate representations that match the feature dimensions of SAM2 outputs. Depth information helps supplement regions that are difficult to accurately distinguish based solely on RGB features, providing a solid foundation for subsequent feature fusion.

**Fig 3:**
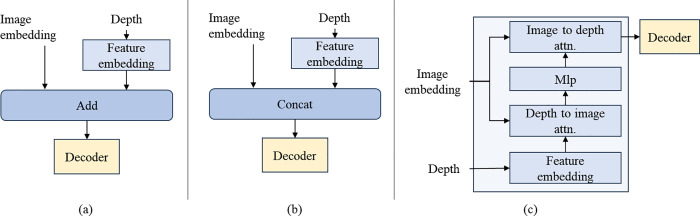
Multi-modal fusion methods, (a) addition; (b) concatenation; (c) cross-attention fusion.


As shown in Fig 3, to maximize the utilization of information from different modalities, we designed a cross-modal cross-attention mechanism to efficiently fuse the visual features from SAM2 with depth information. The inputs are the high-level semantic image features generated by SAM2 and the depth features obtained from the depth network. Typically, modality fusion involves direct addition or concatenation, but these methods do not differentiate the importance of fusion between different modalities. Therefore, we design a multi-modal cross-attention structure. The attention mechanism includes query vectors (*Q*), key vectors (*K*), and value vectors (*V*), and applies a Softmax function to transform the results into attention weight distributions, mathematically defined as follows:

$$\text{Attention}(Q,K,V)=\text{softmax}\left(\frac{QK^{T}}{\sqrt{d_{k}}}\right)V$$ (2)

Where, $d_{k}$ denotes the dimensionality of the keys, Q, K, and V represent the query, key, and value matrices respectively.

Cross-attention sets image features as the query vector and depth features as the key-value vectors, then further extracts the fused features through an MLP. Subsequently, it sets depth features as the query vector and image features as the key-value vectors, dynamically adjusting the attention weights between the two modalities to capture their correlations. This approach ensures attention to the spatial geometric structure of objects while maintaining salient semantic information. The multi-head attention mechanism is adopted to further enhance the ability to capture various feature patterns, improving the fusion effect. Cross-attention effectively combines data from different sources, enhancing the accuracy of salient object localization and the precision of its boundaries.

### 2.4. UNet decoding network based on progressive upsampling

To obtain the final high-resolution saliency object segmentation map, the fused multi-modal features are fed into a decoder based on the U-Net architecture. The key feature of the U-Net decoder lies in its layer-by-layer upsampling operations that gradually restore the spatial resolution of the segmentation map. By using skip connections, it combines high-resolution low-level features from the encoding stage, ensuring that detail information is not lost during the upsampling process. This method effectively captures salient objects across different modalities, thereby achieving high-precision segmentation results. Feature fusion at each layer can be achieved as follows:


$${𝐅}_{\text{skip}\;}=\text{Conv}\left({𝐅}_{\text{low}\;}\right)$$ (3)

$${𝐅}_{\text{final}\;}={𝐅}_{up}+{𝐅}_{skip}$$ (4)

Where, ${𝐅}_{\text{low}\;}$ represents the low-level features from the encoder, ${𝐅}_{up}$ represents the upsampled high-level features, and ${𝐅}_{skip}$ represents the residual skip connection.

### 2.5. Evaluation metrics

To comprehensively evaluate the performance of the model, we adopt Mean Absolute Error (MAE), Maximum F-measure (MaxF), and S-measure (SM) to assess the salient object detection model. MAE measures the pixel-level difference between the predicted saliency map and the ground truth saliency map. F-measure takes into account both Precision and Recall. S-measure combines object-level similarity (S_object) and region-level similarity (S_region) to comprehensively evaluate the quality of the saliency map.

The specific definitions of these evaluation metrics are as follows:


$$\text{MAE}=\frac{\text{1}}{W\times H}\sum_{i=\text{1}}^{W}\;\sum_{j=\text{1}}^{H}\;|S(i,j)-G(i,j)|$$ (5)

Where, *W* and *H* are the width and height of the image, respectively, S(i,j) is the pixel value of the predicted saliency map at position (*i*, *j*), and G(i,j) is the pixel value of the ground truth saliency map at position (*i*, *j*).


$$\text{MaxF}=\underset{\theta }{max}\;\left(\frac{\text{2}\cdot \text{Precision}(\theta )\cdot \text{Recall}(\theta )}{\text{Precision}(\theta )+\text{Recall}(\theta )}\right)$$ (6)

Where, θ is the threshold used for binarizing the predicted saliency map, and Precision(θ) and Recall(θ) are the precision and recall calculated based on the threshold θ.


$$\;\text{S}\text{M}\;=\alpha \cdot {\text{S}}_{\text{object}\;}+(\text{1}-\alpha )\cdot {\text{S}}_{\text{region}\;}$$ (7)

$${\text{S}}_{\text{object}}=max\left(\frac{\mu (S)\cdot Q_{f}}{\mu (G)\cdot Q_{g}},\frac{(\text{1}-\mu (S))\cdot Q_{f}}{(\text{1}-\mu (G))\cdot Q_{g}}\right)$$ (8)

$${\text{S}}_{\text{region}\;}=\lambda \cdot \text{SSIM}\left(S_{f},G_{f}\right)+(\text{1}-\lambda )\cdot \text{SSIM}\left(S_{g},G_{g}\right)$$ (9)

Where, *α* is a balancing parameter, μ(S) and μ(G) are the average saliency values of the predicted and ground truth saliency maps, respectively. $Q_{f}$ and $Q_{g}$ are the quality measures for the foreground and background, respectively. $\text{SSIM}\left(S_{f},G_{f}\right)$ and $\text{SSIM}\left(S_{b},G_{b}\right)$ are the structural similarity indices for the foreground and background regions, respectively, and λ is set to 0.5.

## 3. Results and discussion

This study evaluates the model’s performance in saliency prediction using five popular datasets: DUTS-TE [29], ECSSD [30], PASCAL-S [31], HKU-IS [32], and DUT-OMRON [33]. DUTS-TE contains 5,019 images. ECSSD includes 1,000 images with complex backgrounds and structures. PASCAL-S comprises 850 images with minimal dataset bias. HKU-IS features multiple scattered salient objects across 4,447 images. DUT-OMRON contains 5,168 images of salient objects.

The model was trained using a 3090 GPU, with a learning rate set to 0.001, the AdamW optimizer, a batch size of 12, and a total of 20 training epochs. Input images were resized to 320 × 320, and the training dataset used was DUTS-TR.

### 3.1. Comparative analysis of different methods

Table 1 shows the quantitative results of different models on the DUTS-TE, ECSSD, PASCAL-S, HKU-IS, and DUT-OMRON datasets. Our method is compared with ten advanced models, including NLDF [34], PiCANet [35], CPD [36], PoolNet [37], EGNet [38], SCRN [39], ITSD [40], LDF [41], PFSNet [42], and EDN [43].

**Table 1:** Test metrics of different methods on different datasets.

Method	DUTS-TE		ECSSD		PASCAL-S		HKU-IS		DUT-OMRON	
	MaxF↑	MAE↓	MaxF↑	MAE↓	MaxF↑	MAE↓	MaxF↑	MAE↓	MaxF↑	MAE↓
NLDF	0.813	0.065	0.095	0.063	0.822	0.098	0.902	0.048	0.753	0.080
PiCANet	0.860	0.051	0.935	0.046	0.857	0.076	0.918	0.043	0.803	0.065
CPD	0.865	0.043	0.939	0.037	0.859	0.071	0.925	0.034	0.797	0.056
PoolNet	0.880	0.040	0.944	0.039	0.863	0.075	0.931	0.034	0.808	0.056
EGNet	0.889	0.039	0.947	0.037	0.865	0.074	0.934	0.032	0.815	0.053
SCRN	0.880	0.040	0.950	0.037	0.877	0.063	0.934	0.034	0.811	0.056
ITSD	0.885	0.040	0.946	0.035	0.872	0.065	0.935	0.030	0.821	0.059
LDF	0.898	0.034	0.950	0.034	0.874	0.060	0.939	0.028	0.820	0.052
PFSNet	0.896	0.036	0.952	0.031	0.875	0.063	0.943	0.026	0.823	0.055
EDN	0.895	0.035	0.951	0.032	0.880	0.062	0.941	0.026	0.828	0.049
Our	**0.927**	**0.024**	**0.964**	**0.024**	**0.898**	**0.048**	**0.952**	**0.022**	**0.847**	**0.041**

Compared to the baseline model (EDN), our method demonstrates significant improvements. Specifically, for the MaxF metric, our method outperforms EDN by 3.2%, 1.3%, 1.8%, 1.1%, and 1.9% on the DUTS-TE, ECSSD, PASCAL-S, HKU-IS, and DUT-OMRON datasets, respectively. For the MAE metric, our method reduces the error by 1.1%, 0.8%, 1.4%, 0.4%, and 0.8% relative to EDN on these five datasets.

These results highlight that our method achieves substantial improvements over existing state-of-the-art models, particularly in terms of segmentation accuracy and error reduction.

**Fig 4:**
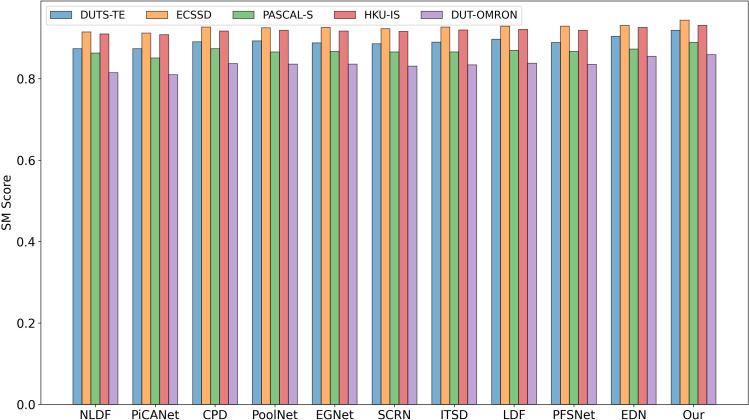
Performance of different methods on the SM metric.

As shown in Fig 4, our method performs excellently on the S-measure (SM) metric, particularly excelling in challenging scenes such as small objects, large objects, and multiple complex objects.

### 3.2. Visualization of results

**Fig 5:**
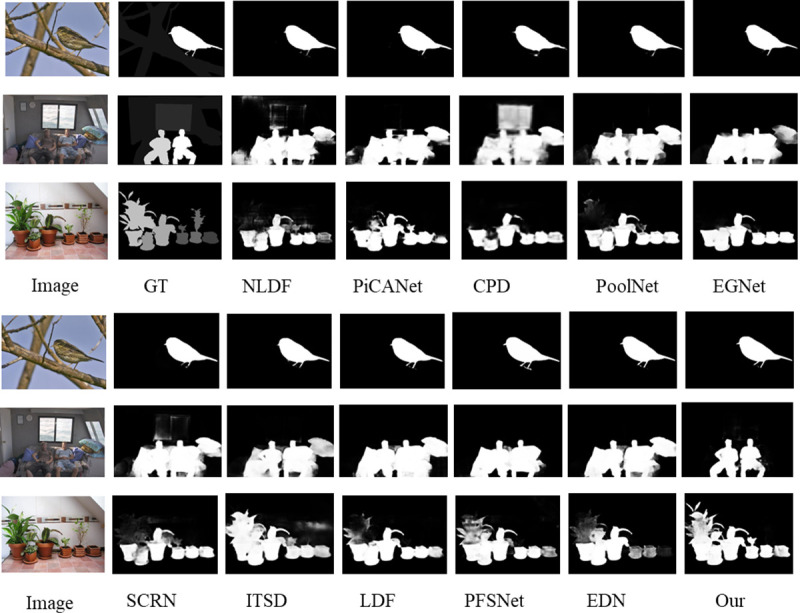
Visualization comparison of prediction results from different methods.

As shown in Fig 5, the proposed method achieves the most accurate overall detection results for salient regions, with more precise and complete target boundaries. Especially in complex scenes and multi-object situations, it can accurately segment salient objects.

### 3.3. Model ablation study

As shown in Table 2, to evaluate the impact of different components on model performance, we conducted ablation studies by progressively adding LoRA (low-rank adaptation), the depth information module, and the cross-attention mechanism. The results indicate that after introducing LoRA, the model’s performance improved, particularly in the MaxF metric. For example, on the DUTS-TE dataset, MaxF increased from 0.879 to 0.891, and MAE decreased from 0.038 to 0.036. This suggests that LoRA helps enhance the model’s generalization ability and reduces error.Further incorporating the depth information module significantly improved the model’s performance across multiple datasets. On the ECSSD dataset, MaxF increased from 0.945 to 0.952, and MAE decreased from 0.034 to 0.029. Notably, on the PASCAL-S dataset, MaxF rose from 0.878 to 0.890, and MAE dropped from 0.062 to 0.057. While the SAM2 model provides a strong foundation, the addition of depth information provided a clear and consistent performance boost. This demonstrates that the additional geometric structure information provided by the depth information module aids the model in better understanding objects within scenes.

**Table 2:** Ablation study of different modules in the model.

Method	DUTS-TE		ECSSD		PASCAL-S		HKU-IS		DUT-OMRON	
	MaxF↑	MAE↓	MaxF↑	MAE↓	MaxF↑	MAE↓	MaxF↑	MAE↓	MaxF↑	MAE↓
Base	0.879	0.038	0.941	0.037	0.871	0.065	0.923	0.034	0.820	0.053
+ LoRA	0.891	0.036	0.945	0.034	0.878	0.062	0.930	0.029	0.831	0.050
+ Depth	0.918	0.032	0.952	0.029	0.890	0.057	0.943	0.025	0.838	0.047
+ Cross-Attention	**0.927**	**0.024**	**0.964**	**0.024**	**0.898**	**0.048**	**0.952**	**0.022**	**0.847**	**0.041**

Finally, introducing the cross-attention mechanism brought the model to its optimal performance. On the HKU-IS dataset, MaxF increased from 0.943 to 0.952, and MAE decreased from 0.025 to 0.022. Particularly on the DUT-OMRON dataset, MaxF rose from 0.838 to 0.847, and MAE decreased from 0.047 to 0.041, enhancing the model’s capability to handle complex scenes, especially in multi-object scenarios for salient object segmentation.

Therefore, the progressive introduction of LoRA, the depth information module, and the cross-attention mechanism significantly boosted the model’s predictive performance. Cumulatively, the addition of both depth and cross-attention yielded significant gains over the LoRA-adapted baseline. While SAM2 is a powerful feature extractor, these results confirm that our proposed depth-guided, cross-attention fusion architecture is not merely refining the results, but is crucial for effectively integrating the multi-modal features to achieve state-of-the-art performance. The synergistic effects among these components not only improved the model’s performance across various datasets but also demonstrated its strong recognition capabilities and robustness. Each added module brought non-linear performance gains, proving the effectiveness and necessity of these techniques. While our ablation study confirms the contribution of each component, future work could explore alternative fusion architectures or conduct a sensitivity analysis on the LoRA rank to further optimize the framework.

**Fig 6:**
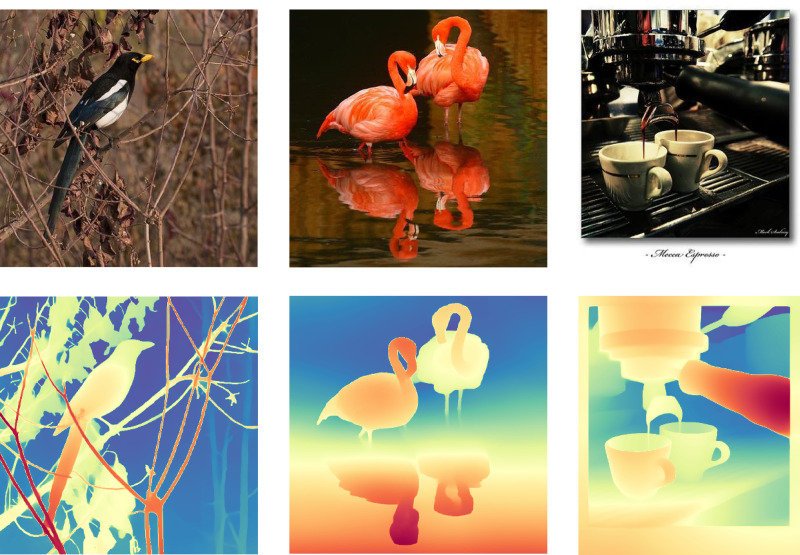
Depth estimation information for different images.

As shown in Fig 6, depth information provides a detailed description of the contents within the image, better illustrating the spatial and depth relationships between objects. This is particularly helpful for multimodal feature combination, especially in complex scenes where it is difficult to identify salient subjects.

## 4. Conclusion

This study introduced an innovative salient object segmentation approach by proposing an effective framework for adapting large foundation models (SAM) using multi-modal guidance. We demonstrated that by integrating SAM’s feature extraction capabilities, geometric enhancements from estimated depth information, and a cross-modal attention mechanism, segmentation performance is significantly improved, particularly in complex scenes involving multiple objects, small targets, and occlusions. The method’s main contributions are as follows:

(1) Cross-Modal Attention Mechanism: We propose a cross-modal attention mechanism that effectively integrates RGB and estimated depth information. Depth data supplements the geometric structure, while the cross-modal attention dynamically adjusts the weight distribution between RGB and depth features. This enhances segmentation performance, especially in complex scenes.
(2) Efficient Training with LoRA: By employing lightweight LoRA fine-tuning, we reduce model training complexity and mitigate overfitting risks while retaining SAM’s versatility and generalization capabilities. This allows for efficient transfer learning tailored to salient object datasets.
(3) Progressive Upsampling Decoding Structure: The proposed UNet-based decoding structure progressively upsamples the feature maps, achieving higher segmentation accuracy and boundary refinement. This design preserves spatial detail information, resulting in high-quality saliency maps.

Through comprehensive evaluation on five benchmark datasets (DUTS-TE, ECSSD, PASCAL-S, HKU-IS, and DUT-OMRON), the method has demonstrated superior performance across multiple key metrics. For instance, on the DUTS-TE dataset, MaxF reached 0.927 and MAE was reduced to 0.024; on the ECSSD dataset, MaxF achieved 0.964 with an MAE of 0.024. The model excels particularly in multi-object, small target, and complex background scenarios, showcasing excellent segmentation performance and robustness.

## References

[1] Zhou T, Fan D-P, Cheng M-M, Shen J, Shao L. RGB-D salient object detection: A survey. Comput Vis Media (Beijing). 2021;7(1):37–69. doi: 10.1007/s41095-020-0199-z 33432275 PMC7788385

[2] Zhuge M, Fan D-P, Liu N, Zhang D, Xu D, Shao L. Salient Object Detection via Integrity Learning. IEEE Trans Pattern Anal Mach Intell. 2023;45(3):3738–52. doi: 10.1109/TPAMI.2022.3179526 35666793

[3] Ji Y, Zhang H, Zhang Z, Liu M. CNN-based encoder-decoder networks for salient object detection: A comprehensive review and recent advances. Information Sciences. 2021;546:835–57. doi: 10.1016/j.ins.2020.09.003

[4] Li G, Bai Z, Liu Z, Zhang X, Ling H. Salient Object Detection in Optical Remote Sensing Images Driven by Transformer. IEEE Trans Image Process. 2023;32:5257–69. doi: 10.1109/TIP.2023.3314285 37721873

[5] Gunduz AB, Taskin B, Yavuz AG, Karsligil ME. A better way of extracting dominant colors using salient objects with semantic segmentation. Engineering Applications of Artificial Intelligence. 2021;100:104204. doi: 10.1016/j.engappai.2021.104204

[6] Uejima T, Niebur E, Etienne-Cummings R. Proto-Object Based Saliency Model With Texture Detection Channel. Front Comput Neurosci. 2020;14:541581. doi: 10.3389/fncom.2020.541581 33071766 PMC7541834

[7] Jiang X, Hou Y, Tian H, Zhu L. Mirror complementary transformer network for RGB‐thermal salient object detection. IET Computer Vision. 2023;18(1):15–32. doi: 10.1049/cvi2.12221

[8] Bruno A, Gugliuzza F, Pirrone R, Ardizzone E. A Multi-Scale Colour and Keypoint Density-Based Approach for Visual Saliency Detection. IEEE Access. 2020;8:121330–43. doi: 10.1109/access.2020.3006700

[9] Wu Z, Su L, Huang Q. Cascaded partial decoder for fast and accurate salient object detection. In: Proceedings of the IEEE/CVF Conference on Computer Vision and Pattern Recognition, 2019. 3907–16.

[10] Shen K, Zhou X, Liu Z. MINet: Multiscale Interactive Network for Real-Time Salient Object Detection of Strip Steel Surface Defects. IEEE Trans Ind Inf. 2024;20(5):7842–52. doi: 10.1109/tii.2024.3366221

[11] Song Z, Sui H, Hua L. A hierarchical object detection method in large-scale optical remote sensing satellite imagery using saliency detection and CNN. International Journal of Remote Sensing. 2021;42(8):2827–47. doi: 10.1080/01431161.2020.1826059

[12] Cheng M-M, Gao S-H, Borji A, Tan Y-Q, Lin Z, Wang M. A Highly Efficient Model to Study the Semantics of Salient Object Detection. IEEE Trans Pattern Anal Mach Intell. 2022;44(11):8006–21. doi: 10.1109/TPAMI.2021.3107956 34437058

[13] Chen Q, Liu Z, Zhang Y, Fu K, Zhao Q, Du H. RGB-D Salient Object Detection via 3D Convolutional Neural Networks. AAAI. 2021;35(2):1063–71. doi: 10.1609/aaai.v35i2.16191 36099219

[14] Han K, Wang Y, Chen H, Chen X, Guo J, Liu Z, et al. A Survey on Vision Transformer. IEEE Trans Pattern Anal Mach Intell. 2023;45(1):87–110. doi: 10.1109/TPAMI.2022.3152247 35180075

[15] Oquab M, Darcet T, Moutakanni T. Dinov2: Learning robust visual features without supervision. arXiv preprint. 2023. doi: 10.48550/arXiv.2304.07193

[16] Liu N, Nan K, Zhao W, Yao X, Han J. Learning Complementary Spatial-Temporal Transformer for Video Salient Object Detection. IEEE Trans Neural Netw Learn Syst. 2024;35(8):10663–73. doi: 10.1109/TNNLS.2023.3243246 37027778

[17] Zheng Q, Zheng L, Deng J, Li Y, Shang C, Shen Q. Transformer-based hierarchical dynamic decoders for salient object detection. Knowledge-Based Systems. 2023;282:111075. doi: 10.1016/j.knosys.2023.111075

[18] Qiu Y, Liu Y, Zhang L, Lu H, Xu J. Boosting Salient Object Detection With Transformer-Based Asymmetric Bilateral U-Net. IEEE Trans Circuits Syst Video Technol. 2024;34(4):2332–45. doi: 10.1109/tcsvt.2023.3307693

[19] Liu Z, Lin Y, Cao Y, et al. Swin transformer: hierarchical vision transformer using shifted windows. In: Proceedings of the IEEE/CVF International Conference on Computer Vision, 2021. 10012–22.

[20] Fan H, Xiong B, Mangalam K. Multiscale vision transformers. In: Proceedings of the IEEE/CVF International Conference on Computer Vision, 2021. 6824–35.

[21] Fu K, Fan D-P, Ji G-P, Zhao Q, Shen J, Zhu C. Siamese Network for RGB-D Salient Object Detection and Beyond. IEEE Trans Pattern Anal Mach Intell. 2021. doi: 10.1109/TPAMI.2021.3073689 33861691

[22] Wen H, Yan C, Zhou X, Cong R, Sun Y, Zheng B, et al. Dynamic Selective Network for RGB-D Salient Object Detection. IEEE Trans Image Process. 2021;30:9179–92. doi: 10.1109/TIP.2021.3123548 34739374

[23] Li C, Cong R, Piao Y. RGB-D salient object detection with cross-modality modulation and selection. In: Computer Vision–ECCV 2020: 16th European Conference, Glasgow, UK, 2020. 225–41.

[24] Sun F, Ren P, Yin B, Wang F, Li H. CATNet: A Cascaded and Aggregated Transformer Network for RGB-D Salient Object Detection. IEEE Trans Multimedia. 2024;26:2249–62. doi: 10.1109/tmm.2023.3294003

[25] Kirillov A, Mintun E, Ravi N. Segment anything. In: Proceedings of the IEEE/CVF International Conference on Computer Vision, 2023. 4015–26.

[26] Ravi N, Gabeur V, Hu YT. Sam 2: Segment anything in images and videos. In: 2024. https://arxiv.org/abs/2408.00714

[27] Ke L, Ye M, Danelljan M. Segment anything in high quality. Advances in Neural Information Processing Systems. 2024;36.

[28] Hu EJ, Shen Y, Wallis P. Lora: Low-rank adaptation of large language models. arXiv preprint. 2021. https://arxiv.org/abs/2106.09685

[29] Wang L, Lu H, Wang Y. Learning to detect salient objects with image-level supervision. In: Proceedings of the IEEE conference on computer vision and pattern recognition, 2017. 136–45.

[30] Yan Q, Xu L, Shi J, Jia J. Hierarchical Saliency Detection. In: 2013 IEEE Conference on Computer Vision and Pattern Recognition, 2013. 1155–62. doi: 10.1109/cvpr.2013.153

[31] Li Y. The secrets of salient object segmentation. In: Proceedings of the IEEE Conference on Computer Vision and Pattern Recognition, 2014.

[32] Guanbin Li, Yu Y. Visual saliency based on multiscale deep features. In: 2015 IEEE Conference on Computer Vision and Pattern Recognition (CVPR), 2015. 5455–63. doi: 10.1109/cvpr.2015.7299184

[33] Yang C, Zhang L, Lu H. Saliency detection via graph-based manifold ranking. In: Proceedings of the IEEE Conference on Computer Vision and Pattern Recognition, 2013. 3166–73.

[34] Luo Z, Mishra A, Achkar A. Non-local deep features for salient object detection. In: Proceedings of the IEEE Conference on Computer Vision and Pattern Recognition, 2017. 6609–17.

[35] Liu N, Han J, Yang M-H. PiCANet: Learning Pixel-Wise Contextual Attention for Saliency Detection. In: 2018 IEEE/CVF Conference on Computer Vision and Pattern Recognition, 2018. 3089–98. doi: 10.1109/cvpr.2018.00326

[36] Wu Z, Su L, Huang Q. Cascaded partial decoder for fast and accurate salient object detection. In: Proceedings of the IEEE/CVF Conference on Computer Vision and Pattern Recognition, 2019. 3907–16.

[37] Liu JJ, Hou Q, Cheng MM. A simple pooling-based design for real-time salient object detection. In: Proceedings of the IEEE/CVF Conference on Computer Vision and Pattern Recognition, 2019. 3917–26.

[38] Zhao JX, Liu JJ, Fan DP. EGNet: Edge guidance network for salient object detection. In: Proceedings of the IEEE/CVF International Conference on Computer Vision, 2019. 8779–88.

[39] Wu Z, Su L, Huang Q. Stacked cross refinement network for edge-aware salient object detection. In: Proceedings of the IEEE/CVF International Conference on Computer Vision, 2019. 7264–73.

[40] Zhou H, Xie X, Lai JH. Interactive two-stream decoder for accurate and fast saliency detection. In: Proceedings of the IEEE/CVF Conference on Computer Vision and Pattern Recognition, 2020. 9141–50.

[41] Wei J, Wang S, Wu Z. Label decoupling framework for salient object detection. In: Proceedings of the IEEE/CVF Conference on Computer Vision and Pattern Recognition, 2020. 13025–34.

[42] Ma M, Xia C, Li J. Pyramidal Feature Shrinking for Salient Object Detection. AAAI. 2021;35(3):2311–8. doi: 10.1609/aaai.v35i3.16331

[43] Wu Y-H, Liu Y, Zhang L, Cheng M-M, Ren B. EDN: Salient Object Detection via Extremely-Downsampled Network. IEEE Trans Image Process. 2022;31:3125–36. doi: 10.1109/TIP.2022.3164550 35412981

